# Blue Laser (450 nm) Treatment of Solar Lentigines

**DOI:** 10.3390/jcm10214919

**Published:** 2021-10-24

**Authors:** Jacek Szymańczyk, Witold Trzeciakowski, Yurij Ivonyak, Piotr Tuchowski, Artem Bercha, Janusz Szymańczyk

**Affiliations:** 1 Department of Dermatology, Medical University of Warsaw, Koszykowa 82A, 02-008 Warsaw, Poland; szymanczyk.jacek@gmail.com; 2 Institute of High Pressure Physics, Polish Academy of Sciences, Sokołowska 29/37, 01-142 Warsaw, Poland; yi@unipress.waw.pl (Y.I.); artem@unipress.waw.pl (A.B.); 3 Accuro Ltd., Zawodzie 16, 02-981 Warsaw, Poland; p.tuchowski@accuro.pl; 4 Department of Dermatology and Venerology, Medical University of Łódź, 90-643 Łódź, Poland; januszsz7@gmail.com

**Keywords:** solar lentigines, blue laser, laser therapy

## Abstract

This study tested a blue light source for the treatment of solar lentigines. A total of 14 patients with solar lentigines were treated with radiation from a novel, high-power 450 nm blue laser that was created for this project. The group contained eight patients with solar lentigines on the face, two patients with the lesions on the dorsal of the hands, and four patients with the lesions on the trunk and forearms. The best results (complete recovery) have been achieved for the lesions on the face and dorsal of the hands. The treatment of lesions on the trunk and forearms was not fully satisfying due to the occurrence of slight scarring. This study shows that, in some cases, the use of a blue laser may be an alternative to the use of longer wavelength sources.

## 1. Introduction

Solar lentigines (LS) are sharply demarcated, hyperpigmented patches that range in size from a few mm in diameter to more than 1 cm [1]. Typically, these lesions are located in areas chronically exposed to sunlight, such as the face, neck, dorsum of the hands, and forearms [2]. These lesions primarily develop in older people, and in the age group over 60 years, they occur in approximately 90% of cases [1]. These lesions, although caused by UV radiation, have no oncogenic potential, and do not require medical intervention. They represent only a cosmetic defect. However, many patients choose to improve their appearance and remove solar hyperpigmentation [3]. As a standard treatment, liquid nitrogen cryotherapy is recommended by the “Pigmentary Disorders Academy” [4]. This method shows good therapeutic results, but patients do not always accept the discomfort during treatment (pain, redness, blister formation, and quite long healing time), as well as the possibility of side effects in the form of post inflammatory hyperpigmentation [5,6]. Cryotherapy is still the most commonly used form of treatment for lentigines, despite these drawbacks. There are more and more reports on the use of various laser devices as well as topical products or chemical peels in the treatment of these conditions [4,7]. There are also many publications describing the positive effects of topical products containing adapalene and a fixed combination of 2% mequinol and 0.01% tretinoine, which are an alternative to ablative methods of treatment [4,8,9,10,11].

Since the early 1960s, the development of laser technology has led to the use of different wavelengths emitted by these devices for the treatment of sun-pigmented lesions. The absorption by melanin in a wide spectral range resulted in attempts to treat lentigines with different lasers [3]. Since the blue laser emission at 450 nm is strongly absorbed by melanin (and its relatively shallow penetration should not be a problem for lentigines), we decided to try this wavelength for the treatment of sun-pigmented lesions. Blue lasers and lights are a hot topic these days in dermatology, and have been proposed in the management of various conditions. The most common application of blue laser is the photodynamic diagnosis and therapy with blue-violet light [12], treatment of actinic keratosis [13], and treatment of lichen sclerosus [14]. Blue light can be used to treat acne [15], reduce sebaceous hyperplasia [16], and stimulate hair regrowth [17]. Our recent studies have shown the positive results of treating Port Wine Stains and telangiectasia with a 450 nm laser [18] and using it as a surgical tool instead of a CO_2_ laser [19]. To our knowledge, a blue laser has not been tested for the treatment of lentigines.

## 2. Materials and Methods

### 2.1. Blue laser Source

Our laser was developed at the Institute of High Pressure Physics for the common research project. It was based on 8 blue laser diodes coupled to a single fiber using the method described in [20]. The laser operates in continuous mode and in pulsed mode (pulse duration from 1 millisecond upwards). The maximum power is 48 W, and the size of the laser spot is adjustable from 0.5 to 5 mm. All treatment parameters are selected on the touch screen, and the device contains a power meter (to enable the measurement of emitted power right before the treatment). More details can be found in [18].

### 2.2. Patients and Treatment Methods

The project received the approval of the Bioethical Commission of the Medical University of Warsaw (Approval Code KB/212/2015). A total of 14 patients with solar lentigines were treated using the 450 nm laser source described above. The treatments started in May 2016 and were completed in September 2020. All clinical work was performed at the Dermatology Clinic of Warsaw Medical University. The patients signed the treatment agreement form and were checked for the following contraindications: pregnant or breastfeeding women, patients with phototoxic or photo-allergic reaction, epilepsy, cancer and precancerous lesions, patients with a strong sunburn, and patients with herpes simplex in the treated area.

Skin type in all patients was evaluated according to the 6-point Fitzpatric scale, i.e., in terms of the skin reaction to UV radiation [21]. The lower the type, the easier it is to burn.

There were 2 patients with the lesions on the dorsal surface of the hands, 2 with lesions on the forearms, 1 patient with lesions on the chest, 1 with lesions on the chest and the forearms, and 8 with lesions on the face. In Table 1, we list the data for all the patients. Irradiations were performed at one-month intervals. In total, there were 28 treatments. Therefore, on average, each patient received 2 treatments. The interval between treatments was typically 1 month, and the second treatment was concentrated on the parts of the lentigo that were not bleached after the first treatment.

The irradiations were carried out using a fiber terminated with a hand-piece with optics of variable focal length, with an adjustable spot diameter from 0.5 mm to 5 mm. During the study, we found that for a spot diameter of 5 mm, the power density was too low, and we therefore decided primarily to use a spot diameter of 2 mm for irradiation. The entire area of the lesion was irradiated during each treatment. We conducted the first irradiations using a power of 4 watts and an exposure time of 50 ms. We then tested higher powers (up to 47 W) and reduced the pulse lengths (down to 15 ms).

The irradiations turned out to be rather painful for the patients, so we used local anesthetic in the form of 5% EMLA (lidocaine and prilocaine) cream and cooled the tissue with a flow of air. The temperature of the air in the cooler was −30 °C, but the typical temperature of cooled skin was 6–8 °C. The local cooling reduced the pain, and the evacuation of heat reduced the risk of scarring.

## 3. Results

**Figure 1:**
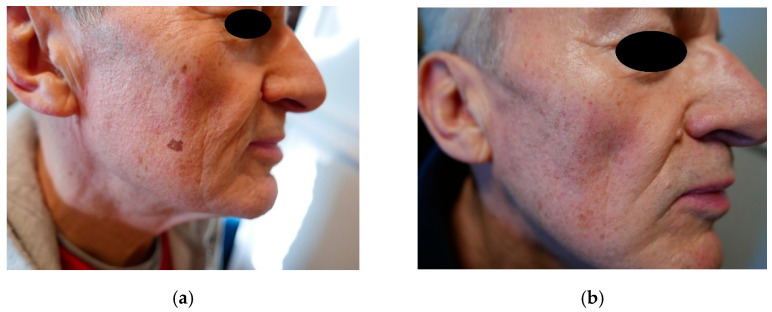
(**a**) Solar lentigines on the face, before treatment, (**b**) solar lentigines on the face, after treatment.

**Figure 2:**
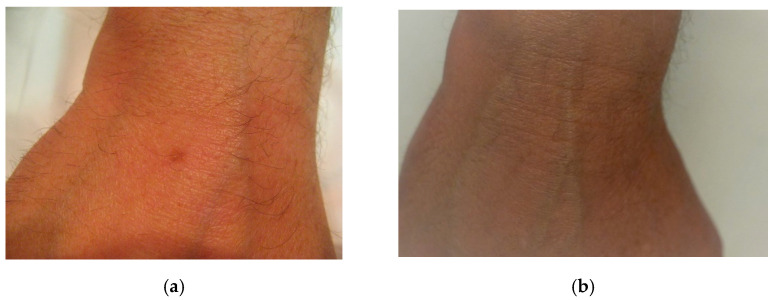
(**a**) Solar lentigines on the hand, before treatment, (**b**) solar lentigines on the hand, after treatment.

**Figure 3:**
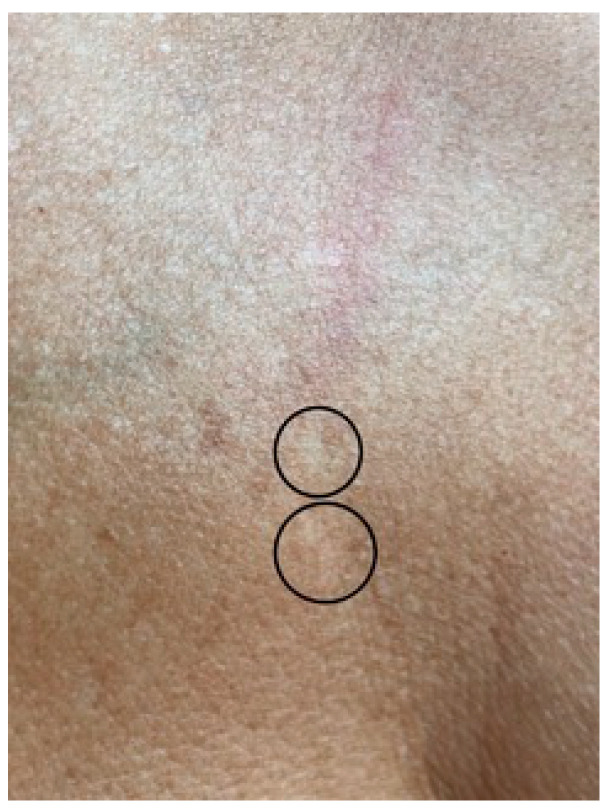
Slight scarring on the chest after laser therapy.

The best cosmetic results in the form of the complete disappearance of lesions were achieved in patients with lesions located on the face (Figure 1a,b) and on the dorsum of the hands (Figure 2a,b). In the case of forearm and décolletage lesions, the treatment was not satisfactory, and in the case of décolletage lesions, the treatment resulted in the formation of small scars (Figure 3). During the tests of irradiation with different power and pulse duration, it turned out that the best results were obtained for 47 W power and a pulse duration of 15 ms. One patient with lesions on the face and neck, after the first irradiation with a power of 9 W and a pulse duration of 200 ms, abandoned further attempts of treatment because of the subjectively assessed, unsatisfactory effect of the treatment. 

## 4. Discussion

The available modalities for the treatment of lentigines boil down to two therapeutic methods: pharmacological and physical. Examples of pharmacological therapies include the use of a high concentration of hydroquinone, hydroxyl acid, and tretinoin-containing products [8,9,10,11,22]. These therapies are often unsatisfactory for patients, require a long treatment period, and often cause skin irritation [23]. Physical therapy methods include cryotherapy, laser therapy, and the use of chemical peels. These methods are much more popular due to the shorter procedure time [24]. Laser devices such as Pulsed Dye (510 or 595 nm) [25], Frequency-Doubled Nd:YAG (532 nm) [24,26,27,28], Q-switched ruby (694 nm) [3,28], Q-switched alexandrite (755 nm) [22], and CO_2_ (10,600 nm) [26] have been used in the treatment of lentigines. All Q-switched (QS) lasers have been shown to be safe and effective in the treatment of these lesions [3]. A 675 nm laser has also been proposed in the management of superficial hyperpigmentation [29].

The most commonly used laser device for the treatment of lentigines today is the frequency doubled Q-switched Nd:YAG laser [24,26,27,28]. Picosecond Q-switched Nd:YAG lasers seem to give a better result in managing lentigo than old nanosecond lasers due to their ability to concentrate energy in minimal fractions of time [30]. Many clinical studies demonstrate the superiority and better therapeutic effects of Nd:YAG over other laser devices, cryotherapy, as well as chemical peels [6,27,28]. In cases using more aggressive settings of the device, however, post-inflammatory hyperpigmentation and longer-lasting redness at the application site may occur, especially in patients with skin type III-IV, according to the Fitzpatric scale (Fitzpatric skin type III-IV) [26,28]. 

Comparable therapeutic effects in the treatment of lentigines as with the use of the Q-switched KTP 532 nm laser can be obtained by using an IPL device with a wavelength filter [22,31].

In most available publications, very good therapeutic results have been obtained in the cases of treatment of lentigines located in areas exposed to the sun, i.e., the face and dorsum of the hands. Attempts to treat lesions located on the upper extremities and trunk show poor efficacy, while attempts to irradiate lentigines located on the lower extremities have failed completely [25]. 

## 5. Conclusions

- The 450 nm blue laser has proven to be another very effective device in the treatment of lentigines. The irradiation parameters were 47 W pulses with a 15 ms duration and 2 mm spot size. Based on results for other lasers (like the KTP laser at 532 nm) we expect that further reduction in the pulse duration (with an increase in peak power) may reduce the risk of scarring.
- In areas where the skin is more delicate, i.e., the décolleté and forearms, the treatment should be performed using less power and shorter exposure time because of the possibility of slight scarring.
- At a 450 nm wavelength, we can expect strong absorption by hemoglobin. However, this absorption peaks around 420 nm and drops for longer wavelengths. It would likely be better to use longer blue wavelengths (like 460–470 nm) to target melanin, and shorter blue wavelengths (like 420 nm) to target hemoglobin. A strong absorption implies shallow penetration in the tissue, so the epidermis (melanocytes) may receive a higher dose of light than dermis (blood vessels). We obtained positive results on the face (thin epidermis) and on the dorsum of the hands (thick epidermis) and scarring on the chest (where the thickness of the epidermis is intermediate), so we have not observed any correlation of results with the thickness of the epidermis. More prospective clinical studies are necessary to confirm the results we obtained with this small pilot study.

## Figures and Tables

**Table 1:** Data for the patients with solar lentigines.

Patient’s Number	Sex	Age	Type of Skin	Location of Lesion	Number of Procedures	Size (mm)	Improve-Ment (%)	Evaluation of Effects
1	F	74	II	Forearms	2	10–20	30	Unsatisfactory
2	F	61	II	Forearms	1	13	30	Unsatisfactory
3	F	72	III	Face (forehead)	6	14–16	100	Complete disappearance
4	F	56	III	Face and neck	1	7–11	0	Resignation after one procedure
5	F	55	IV	Face (forehead and cheek)	3	20	100	Complete disappearance
6	F	52	III	Forearms and chest	2	4–5	100 (forearms)	Scarring on the chest
7	F	62	III	Face (nose)	1	15	100	Complete disappearance
8	F	74	II	Face	1	20	100	Complete disappearance
9	M	70	II	Face (cheek)	3	10	100	Complete disappearance
10	M	68	II	Face (forehead)	2	20	100	Complete disappearance
11	M	61	III	Dorsal of hands	1	5	100	Complete disappearance
12	F	70	II	Chest	1	8	50	Partial disappearance
13	F	60	II	Face (nose)	1	12	50	Partial disappearance
14	M	56	V	Dorsal of hands	3	4–5	100	Complete disappearance

## Data Availability

The data presented in this study are available in Table 1.
